# Evolving perspectives on speech perception assessment in adults with cochlear implants: Are we using the right tests?

**DOI:** 10.3389/fnins.2025.1667467

**Published:** 2025-09-16

**Authors:** Valeriy Shafiro, Aaron C. Moberly, David B. Pisoni, Terrin N. Tamati

**Affiliations:** ^1^Department of Communication Disorders and Sciences, Rush University, Chicago, IL, United States; ^2^Department of Otolaryngology – Head and Neck Surgery, Vanderbilt University Medical Center, Nashville, TN, United States; ^3^Department of Psychological and Brain Sciences, Indiana University, Bloomington, IN, United States; ^4^Department of Otolaryngology-Head and Neck Surgery, Indiana University School of Medicine, Indianapolis, IN, United States

**Keywords:** cochlear implants, speech perception assessment, minimum speech test battery, cochlear implant candidacy, aural rehabilitation, test battery

## Abstract

From the pioneering days of cochlear implants (CIs) more than half a century ago until the present time, speech perception outcomes remain a broadly recognized benchmark of CI success. However, speech test scores alone do not directly map onto individual patients’ aural communication needs. Rather, speech tests provide a relatively time-efficient way to assess specific aspects of everyday speech processing abilities. We review how speech perception testing has evolved in the United States of America since the early days of CIs and critically examine its current clinical roles: (1) establishing CI candidacy, (2) measuring benefit post-CI, and (3) pinpointing specific perceptual deficits to guide counseling, rehabilitation, or programming changes. We further consider: (a) factors that have driven changes in how speech perception has been evaluated over time, (b) approaches to selecting outcome measures of speech perception and interpretation of outcomes, (c) the role that speech perception plays in the assessment of overall CI benefit and individualized rehabilitation, and (d) how test selection and conditions can influence CI care. We argue that conventional speech perception tests provide only a partial view of CI outcomes and call for more comprehensive, ecologically meaningful assessment approaches. We conclude with recommendations for selecting outcome measures that better reflect real-world communication demands and guide patient-centered care for adult CI users.

## 1 Introduction

Speech perception testing is an integral component of the patient’s cochlear implant (CI) journey from an initial evaluation to establish candidacy to post-implantation assessment and counseling to optimize benefit. In this article, we begin by considering speech perception as it refers primarily to clinical assessments commonly used to evaluate spoken word or sentence recognition, and later argue for expanding this approach to better reflect real-world communication. The history of CIs as the first highly successful electronic prosthesis for sensorineural hearing loss (SNHL) cannot be told without persistent references to the results of various speech perception tests used to demonstrate CI benefits. Nevertheless, since the early days of CIs more than half a century ago until now, assessment of speech perception in adult CI users has continued to evolve. Although changes in clinical testing have been rather slow to implement, they have been driven by a variety of separate but interrelated factors. These include the advancements in CI technology and improved patient outcomes, changes in clinical practices and candidacy criteria, as well as expanding theoretical perspectives on speech communication in people with hearing loss. A better understanding of how these factors have influenced speech perception assessment in CI users can assist clinicians and researchers in selecting and interpreting results of specific tests for better alignment with treatment goals of individual patients and a continued improvement in CI outcomes. After briefly reviewing the changes in speech perception testing of CI users over time, we provide specific considerations and potential criteria that can assist CI clinicians and researchers in selecting or developing appropriate tests for outcome measures in clinical and research contexts.

## 2 Early days of cochlear implants

In the 1960s and 1970s, when restoration of functional human hearing by means of electrical stimulation of the auditory nerve was a novel and controversial approach to the treatment of profound hearing loss in adults, the benchmarks for CI success were quite modest by today’s standards (House, 2011). A mere auditory awareness of speech, along with other environmental sounds, was sufficient to demonstrate the promise of the new approach for individuals with profound SNHL using single-channel devices. A noted speech perception benefit demonstrated by many single-channel CI users was an improvement in lipreading scores following implantation. However, without visual cues, open set monosyllabic word scores typically remained near chance (Bilger and Black, 1977; Owens et al., 1985; Wilson and Dorman, 2008).

**Figure 1:**
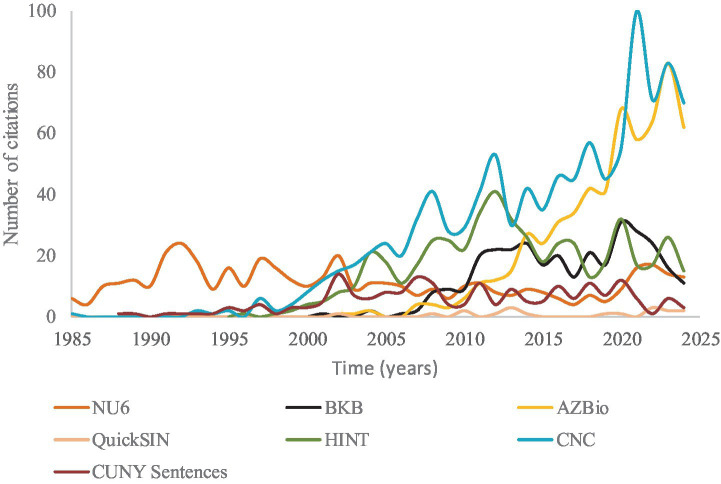
Number of citations (y-axis) per year obtained in Google Scholar for seven common tests used to assess speech perception accuracy in adult cochlear implant (CI) users. NU6, Northwestern University Auditory Test No. 6; QuickSIN, Quick Speech in Noise Test; City University of New York Sentences, CUNY Sentences; BKB, Bamford-Kowal-Bench Speech in Noise Test; HINT, Hearing in Noise Test; AzBio, AzBio Sentences; CNC=Consonant-Nucleus-Consonant monosyllabic words.

As CI technology continued to develop, particularly with the introduction of multi-channel CIs, patients began achieving better speech perception outcomes, creating a need for a more comprehensive assessment of speech perception in patients with varying ability levels, with an added emphasis on testing in the auditory-only condition. Development of new tests was especially important because available monosyllabic word lists alone, which were based on materials developed during World War II to improve electronic telecommunications (Egan, 1948; Hirsh, 1947; Lehiste and Peterson, 1959; Bilger, 1984; Pisoni, 2021), did not have contextual cues and were still too challenging for many CI users. Open-set speech recognition tests also did not provide useful information for optimizing programing strategy, monitoring performance over time or guiding rehabilitation. Additional tests were needed to expand the range of speech perception skills assessed in CI users. Subsequently, the Minimum Auditory Capability (MAC) battery was developed beginning in 1976 and revised during the following decade (Owens et al., 1985) to standardize performance evaluation across CI centers and provide a more comprehensive set of assessment instruments. The MAC battery consisted of 13 auditory-only and one lip-reading tests, with all but one (familiar environmental sounds) of these tests focused on various aspects of speech perception. The tests varied in difficulty from simple discrimination of speech vs. noise to word intelligibility with different amounts of context. The battery also included prosodic tasks such as discriminating questions from statements and identifying word stress. Although the MAC battery was not often administered in full due to the length of time required (2–3 h), some tests from the MAC battery became widely adopted as benchmarks of CI performance and remain so to this day. For instance, the Consonant-Nucleus-Consonant (CNC) word recognition test, first published by Lehiste and Peterson (1959), which drew on the materials from Egan (1948), is currently still the most widely cited test used to characterized speech performance in adults with CIs (Figure 1). Importantly, the MAC tests further highlighted some inconsistencies between CI recipients’ pure tone thresholds and their ability to perceive speech. With multi-channel implants, CI-aided pure tone thresholds could often be obtained in the normal or mild hearing loss range, while most patients still struggled to understand speech presented without visual cues, particularly in noisy backgrounds.

## 3 Speech perception testing beyond the MAC

### 3.1 Establishing expectations and CI candidacy, monitoring outcomes

As device technology evolved and outcomes continued to improve, it became clear that new measures were once again needed both to evaluate outcomes of CI recipients and also to better determine which patients should be considered CI candidates. Since 1985, the United States Food and Drug Administration (FDA) has progressively expanded adult indications for CI beyond bilateral profound SNHL with 0% open set sentence recognition. For example, even as early as 2005, official adult CI candidacy indications (Centers for Medicare and Medicaid Services, 2005) consisted of bilateral, profound SNHL with limited benefit from hearing aid amplification, and scores of 50% or worse words correct on open-set sentence recognition in the ear to be implanted, and worse than 60% in the binaural best-aided configuration.

The original Minimum Speech Test Battery (MSTB) was introduced in 1996 by Nilsson et al. (1996) to provide a standardized set of speech perception tests to be used clinically for adult CI recipients, with input from professional organizations and CI manufacturers. The original MSTB for adults consisted of CNC word recognition and HINT sentences in quiet and noise (Nilsson et al., 1996). The CNC stimuli (Peterson and Lehiste, 1962) were lists of monosyllabic words with equal phonemic distribution across lists, with each list exhibiting approximately the same phonemic distribution as the English language (Lehiste and Peterson, 1959). For open-set sentence recognition, sentences from the Hearing in Noise Test (HINT; Nilsson et al., 1996) were recommended in quiet as well as in speech-spectrum noise. The HINT sentences were meaningful, simple sentences spoken by a single male talker in a clear speaking style. Compact disc (CD) recordings of these tests were generated, distributed, and extensively used across CI centers in the United States (Nilsson et al., 1996).

A second version of the MSTB (“New MSTB” or here referred to as the “MSTB-2”; Advanced Bionics LLC, Cochlear Americas, MED-EL Corporation, 2011) was released in 2011, in response to advances in CI technology and overall improvements in outcomes, along with broadening of CI candidacy criteria. These changes resulted from ceiling effects on HINT sentences in quiet in as many as 71% of adult CI users (Gifford et al., 2008), limiting the test’s utility in capturing persistent variability in CI outcomes and representing real-world speech perception abilities for which CI users still reported difficulties (e.g., speech perception for more complex speech materials or in noisy backgrounds). As a result of these ceiling effects, the MSTB-2 shifted to the use of CNC words and more difficult sentence materials such as the AzBio sentences (Spahr and Dorman, 2004) and the Bamford-Kowal-Bamford Speech-in-Noise (BKB-SIN) test (Etymotic, 2005; Gifford et al., 2010). The AzBio sentences introduced greater variability and difficulty by incorporating two male and two female talkers speaking in a conversational style with limited contextual cues, with sentences ranging in length from four to 12 words. For the MSTB-2, it was recommended to present AzBio sentences in quiet or in 10-talker multi-talker babble at a fixed signal-to-noise ratio (SNR), typically +10 or +5 dB. In contrast, the BKB-SIN test used sentences spoken by a male talker with a modified adaptive approach in which sentences were presented at a fixed level and four-talker babble was presented at increasingly more difficult SNRs in 3-dB SNR steps. Results of the BKB-SIN were reported as the SNR in dB at which the patient could repeat 50% of the key words in the sentences (the SNR-50). A comparison of the SNR-50 of the patient, compared with the SNR-50 of normal-hearing individuals, was computed as the “SNR loss,” representing the degree of difficulty for that patient when listening to speech in noise.

More recently, a third version of the MSTB (“MSTB-3”; Dunn et al., 2024) was developed using a more rigorous modified Delphi consensus process (Rosenfeld et al., 2015). The MSTB-3 was developed to provide updated practice guidelines for clinicians due to further technological advances and surgical approaches resulting in hearing preservation, including expansion of FDA indications. Expansions of CI candidacy have included patients with significant residual hearing who could benefit from electric-acoustic stimulation (EAS), along with individuals with single-sided deafness (SSD) or asymmetric hearing loss (AHL) who often perform at ceiling when tested in binaural best-aided conditions. Although candidacy expansion to these indications had been performed off-label (Carlson, 2020), FDA approval for these indications has occurred in the past few years across CI manufacturers. The MSTB-3 also incorporates patient-reported outcome measures (PROMs), which were not previously included in the MSTB battery.

An important update incorporated into the MSTB-3 is another attempt to address concerns regarding ceiling effects demonstrated by many of today’s CI users (Gifford et al., 2010). In order to avoid ceiling effects, many clinicians will test pre- and/or post-CI users in varying degrees of background noise (typically multi-talker babble; Dunn et al., 2020). However, without standardized protocols, this approach has led to inconsistency among CI centers and even among individual clinicians within CI centers. The MSTB-3 attempts to provide standardization in CI assessment across centers, while also promoting clinical testing efficiency. The MSTB-3 contains recommendations for calibration, and it also provides recommendations for unaided audiometric testing and hearing aid verification prior to speech perception testing, as well as support for streamlining post-CI timing of assessments (e.g., removal of a 6-month post-activation appointment if the patient is obtaining appropriate benefit at 3 months of CI use). Here, however, we will focus on the speech perception tests comprising the MSTB-3.

The primary focus of the MSTB-3 speech perception measures is to serve as a recommendation of test materials for, first, determination of CI candidacy and the recommended ear for implantation. This determination takes into consideration (based on unaided thresholds) which “category” a patient falls into: traditional CI candidate with bilateral moderate-to-profound SNHL, EAS candidate, or SSD or AHL candidate. Second, the MSTB-3 is used for evaluation of the benefit provided by the CI by comparing post-CI versus pre-CI performance. Third, the test battery is used for assessment of performance in ear-specific (e.g., CI-only, hearing aid-only) as well as everyday listening configurations (bilateral testing with CI and hearing aid or two CIs for bilateral CI users). Additional goals of the MSTB-3 beyond CI candidacy and CI outcomes, are to use these measures to monitor the non-implanted ear for eventual CI candidacy and assessment of bimodal benefit (i.e., benefit obtained with a hearing aid on the ear contralateral to the CI), evaluation of performance with other forms of hearing technology beyond CIs, and determination if assistive listening devices or noise suppression strategies may be helpful for individual patients. Together, these efforts represent a broader shift toward individualized, longitudinal assessment of CI performance.

For CI candidacy testing, the MSTB-3 emphasizes the use of CNC word recognition testing, presented at 60 dBA at zero degrees azimuth in the patient’s “best-aided” listening configuration for each ear. With the MSTB-3, “best-aided” has been clarified to refer to the score for an individual ear when the patient is using a hearing aid optimized for the hearing loss in that ear. Advantages of focusing on CNC scores, rather than open-set sentence recognition, are that CNCs are less likely to demonstrate post-operative ceiling effects than sentences (Gifford et al., 2008), and that no semantic context cues are available with words compared with sentences, potentially minimizing top-down influences in order to reveal subtle auditory deficits (Sladen et al., 2017). Moreover, the MSTB-3 places greater emphasis than the prior MSTB batteries on the ear to be implanted, which is more appropriate for determining candidacy in patients with SSD and AHL. Importantly, however, the MSTB-3 does not provide recommendations regarding specific CNC word scores that should be used by CI centers to determine CI candidacy, with the literature providing evidence for appropriate pre-operative CNC candidacy scores ranging from 40–60% word recognition (Sladen et al., 2017; Dunn et al., 2024; Perkins et al., 2021).

If a patient is deemed a CI candidate, based on the CNC score and consideration of medical issues, a recorded list of AzBio sentences (Spahr et al., 2012) is administered in the best-aided condition to the ear to be implanted, using a + 10 dB SNR, with speech and noise at zero degrees azimuth. Clinicians may additionally choose to administer additional AzBio sentences in quiet or at +5 dB SNR to further evaluate the patient’s best-aided hearing or to determine if the patient meets insurance criteria for implantation. Additional test configurations for CNC words and AzBio sentences may be administered as deemed useful for determination by each CI center for CI candidacy recommendations.

After cochlear implantation, the MSTB-3 recommends testing with CNC words, again at 60 dBA at zero degrees azimuth, in the implanted ear. The post-operative test battery also typically includes AzBio sentence testing at +10 dB SNR and/or in quiet in the patient’s “everyday” listening configuration, meaning the patient’s typical binaural listening configuration (e.g., bimodal, bilateral CI, or EAS in one ear and hearing aid in the other ear). Depending on performance, AzBio sentence testing at +5 dB SNR can be administered. For SSD or AHL patients, sentence testing may be performed with spatial separation of the speech and noise, typically using multitalker babble presented at 90 degrees azimuth on the side of the non-implanted ear. Lastly, depending on the patient’s hearing category, monitoring of the contralateral ear may continue using CNC words in the other ear and/or AzBio sentences in the everyday listening configuration.

### 3.2 Alternative and new approaches for assessing outcomes for CI optimization, rehabilitation, and counseling guidance

In addition to the tests included in the current and earlier versions of the MSTB, many clinics and research groups have utilized alternative speech perception tests for characterizing CI speech perception performance (Figure 1). These tests might have been deemed more appropriate for addressing additional aspects of speech processing, enabled comparisons with other populations of adults with hearing loss, or were preferred by the clinical teams based on their prior experience. For instance, City University of New York (CUNY) sentences (Boothroyd et al., 1985) provide an opportunity to supplement auditory-only with visual lip-reading scores. Another test, QuickSIN (Etymotic, 1993; Killion et al., 2004), is similar to BKB SIN in its administration and scoring, but the sentence materials are less predictable semantically, making the test more challenging. Finally, NU6 (Tillman and Carhart, 1966), a monosyllabic word test, similar to CNC, was part of the original MAC battery, but later became less commonly used in CI testing. Overall, the choice of the tests has always remained the responsibility of individual clinicians and CI clinics based on specific settings, patient population and existing protocols.

In general, when interpreting results of any speech recognition test, it is important to keep in mind that test scores can provide clinicians with a basic measure of how well CI users recognize speech sounds under controlled listening conditions. However, they give only a coarse-grained snapshot of communication challenges faced by individual patients in daily life. When test results change, for example as a result of implantation, programming changes or a rehabilitation program, it is often unclear what constitutes a minimal clinically significant difference for an individual patient on a specific test. Thus, interpreting minor test score changes and translating test results into actionable guidance for rehabilitation and counseling remains a persistent challenge. This difficulty stems in part from the disconnect between a small set of controlled test materials typically used in the clinics and the dynamic, social, and often adverse nature of real-world speech communication. Additionally, these tests were not developed to capture meaningful individual differences in real-world speech perception skills by engaging the processing strategies individuals use to understand speech in everyday life. This disconnect emphasizes a major concern of conventional speech recognition testing: while the MSTB-3 and similar tests provide clinical benchmarks, they fall short in evaluating how CI users actually function in their everyday communication environments, underscoring the need for complementary assessments. Key opportunities moving forward are to apply tools from psycholinguistics and cognitive hearing sciences, PROMs, and remote testing to guide personalized rehabilitation and counseling strategies that align with each patient’s unique lifestyle and communication needs.

## 4 Broadening outcome measures

As discussed above, clinical assessment of outcomes in adult CI users continue to rely on isolated word and sentence recognition tests conducted in quiet or fixed noise levels, using highly controlled materials. It should be noted that these measures exhibit several useful features – they are easy to score, are sensitive to degradation (e.g., noise, hearing loss), and also demonstrate changes following implantation (e.g., Baese-Berk et al., 2023). Indeed, many postlingually deafened adult CI users achieve reasonably good open-set speech recognition on conventional clinical speech recognition tests and show improvements up until around 3 months post-CI on average (Ma et al., 2023). Nonetheless, these tests offer only a limited view of the broader real-world experiences and challenges faced by adult CI users. Conventional testing materials typically contain carefully articulated, linguistically simple materials produced by a single talker or a small number of talkers. Additionally, these measures are focused solely on the listener’s ability to recognize isolated words or words in sentences. These test attributes differ from real-world speech communications, which involve dynamic understanding of utterance meaning within specific situational context, often within short time windows of an ongoing back-and-forth conversation and response planning.

There is a need for more comprehensive, ecologically valid assessments that not only measure speech recognition in controlled settings but also capture the cognitive and communicative demands of real-world listening environments. While many tests provide important clinical benchmarks, they do not necessarily predict how CI users will perform in more dynamic, real-world settings. For example, speech recognition in quiet does not necessarily predict success in complex, multi-talker environments where CI users must rapidly adapt to new voices, extract meaning from acoustically variable speech, and manage cognitive load (e.g., Tamati et al., 2023). CI users often rely on visual cues to supplement auditory input. Therefore, assessments that integrate both modalities may provide a more ecologically valid measure of real-world communication ability. The importance of visual cues in speech perception became particularly apparent during the recent COVID-19 pandemic, when masks covering speakers’ mouths made speech perception especially challenging for all people with hearing loss (e.g., Homans and Vroegop, 2021; Sönnichsen et al., 2022). Further, standard clinical tests fail to account for the substantial individual differences in how CI users adapt to real-world challenges (e.g., Moberly et al., 2025; Tamati et al., 2023). For instance, the PRESTO sentence test was specifically developed to reflect the talker and linguistic variability present in everyday listening environments. It also captures meaningful individual differences in the ability to rapidly adapt to such variability (Gilbert et al., 2013; Tamati et al., 2013). Thus, assessments that incorporate real-world complexity, such as audio-visual input, speech originating from multiple talkers, or speech in the presence of competing talkers, would provide a more ecologically valid assessment of CI users’ communicative abilities.

Conventional speech recognition tests provide endpoint “product” measures of performance, offering limited insight into the underlying cognitive processes that support successful communication (Moberly et al., 2018). In addition to the limitations of convention speech recognition measures described above, these measures were not developed to explain or identify the factors underlying the variability in outcomes or address individual differences (Pisoni et al., 2008). When a patient does well after receiving the implant he and his care team are satisfied with the outcome. However, when a patient’s performance does not improve, they are not satisfied, and it is often difficult to explain the reasons or find ways to improve performance. It is thus critical to consider factors that are responsible for individual differences, along with the associated behavioral and neurocognitive domains. To that end, “process” measures assess the underlying cognitive mechanisms that contribute to speech perception. These include information processing speed, working memory, and inhibitory control. Assessing these domains using non-auditory cognitive tasks allows researchers to disentangle cognitive contributions from audibility. For example, our recent work demonstrated that speech recognition scores in adult CI users are associated with working memory, nonverbal reasoning, and lexical access speed (Mattingly et al., 2018; Moberly et al., 2016, 2021; Tamati et al., 2020, 2023). These findings demonstrate how cognitive-linguistic abilities contribute to individual differences in speech perception performance.

Assessments should also consider the cognitive demands of listening in complex environments, as well as the compensatory strategies CI users use to manage listening effort in their daily lives. For example, previous research has shown that even CI users who achieve high accuracy on conventional speech recognition tests may still expend significant cognitive effort to understand speech, particularly in challenging environments (Patel et al., 2023; Winn and Teece, 2021, 2022). Measures such as pupillometry and reaction time tasks are used to assess cognitive demand, providing a more comprehensive picture of the listening experience beyond speech recognition accuracy. For example, a recent study by our group (Patel et al., 2023) demonstrated that CI users experience increased listening effort when comprehending sentences produced by specific talkers, despite showing similar levels of comprehension accuracy across talkers. Further, talker-specific effects were potentially related to the talker’s idiosyncratic speaking style or rate. These findings suggest that talker differences may impose additional cognitive demands, even when speech comprehension appears stable. Together, this growing body of literature highlights a limitation of conventional speech recognition tests: they may obscure underlying effort or variability in information processing, leading to an incomplete picture of CI outcomes. Understanding patterns of listening effort may help guide personalized interventions aimed at improving real-world listening experiences.

### 4.1 Patient-reported outcome measures

The broader functional outcomes associated with CI use have been recently documented using PROMs. In particular, measures of hearing-related quality of life provide insight into CI users’ perception of their real-world communication challenges. They also capture the associated social and emotional impacts of hearing loss. Perhaps not surprisingly, previous research has found weak or inconsistent relationships between performance on conventional clinical speech recognition measures and self-reported real-world hearing-related functioning on PROMs in adult CI users (Brumer et al., 2022; McRackan et al., 2018; Vasil et al., 2020). These findings highlight both the limitations of conventional speech recognition tests and the need for assessment tools that better reflect the demands of functional speech communication in complex, everyday environments, along with the patient’s own appreciation of CI benefits.

PROMs provide valuable insight into aspects of CI users’ self-perceived communication challenges and hearing-related quality of life that extend beyond basic speech recognition. Instruments such as the Cochlear Implant Quality of Life (CIQOL-35) questionnaire (McRackan et al., 2022; McRackan et al., 2019), the Speech, Spatial, and Qualities of Hearing Scale (SSQ; Gatehouse and Noble, 2004), the Vanderbilt Fatigue Scale (VFS-A; Hornsby et al., 2021), and the Listening Effort Questionnaire for Cochlear Implant users (LEQCI; Hughes et al., 2019) have been developed to assess the real-world impacts of hearing loss and cochlear implantation on communication. These PROMs complement behavioral assessments by capturing factors such as social participation, cognitive fatigue, and emotional responses to communication difficulties. Together, PROMs and ecologically meaningful behavioral measures can offer a more comprehensive and clinically meaningful understanding of a patient’s functional communication ability.

### 4.2 New directions

#### 4.2.1 Comprehensive behavioral and patient-reported batteries

To better understand CI users’ real-world communication abilities, researchers have begun to develop more comprehensive assessment batteries that extend beyond conventional speech perception tests. These batteries incorporate not only clinical analogs but also novel speech perception tests as well as a range of auditory and cognitive-linguistic tasks to capture the everyday demands of communication (Moberly et al., 2025; Shafiro et al., 2020; Tamati et al., 2020). For example, we routinely use a comprehensive battery of speech perception (e.g., word and sentence recognition, indexical (talker) perception, PROMs), auditory (e.g., spectral and temporal resolution), and cognitive-linguistic measures (e.g., working memory capacity, inhibitory control, lexical access speed) in our in-person research testing protocols for adult CI users. This comprehensive battery allows us to better define communication outcomes, capture meaningful individual differences, and have a broad base of measures to predict outcomes (e.g., Moberly et al., 2025; Patro et al., 2024; Tamati et al., 2020).

Remote assessment has also become an increasingly valuable approach for assessing outcomes in adult CI users since it enables access to participants outside of clinical settings and facilitates large-scale data collection (Peng et al., 2022). The BASE (Basic Auditory Skills Evaluation) battery, developed by Shafiro et al. (2020),^1^ is an example of a comprehensive remote battery for evaluating auditory perception in CI users. This battery includes tests that assess spectro-temporal processing, speech recognition in quiet and in-noise, indexical (talker) perception and environmental sound perception tests with a variable working memory load. Other compilations of tests for the assessment of speech perception along with a wide range of other auditory abilities can be found on TeamHearing.org as well as Portable Automated Rapid Testing (PART; Lelo de Larrea-Mancera et al., 2022). Building on this approach further, recent work by Tamati and colleagues has developed a fully remote battery that integrates both behavioral measures and PROMs, tailored specifically to real-world communication and cognitive effort (Tamati et al., 2024). This battery includes tasks such as sentence comprehension, recognition memory, and indexical (talker) processing tasks, alongside PROMs that capture self-reported hearing-related quality of life, listening effort and fatigue, and communication challenges. Initial studies demonstrate the feasibility of using this remote battery. Importantly, both batteries are designed for remote administration, which enables broader research participation by reducing geographic and logistical barriers.

**Figure 2:**
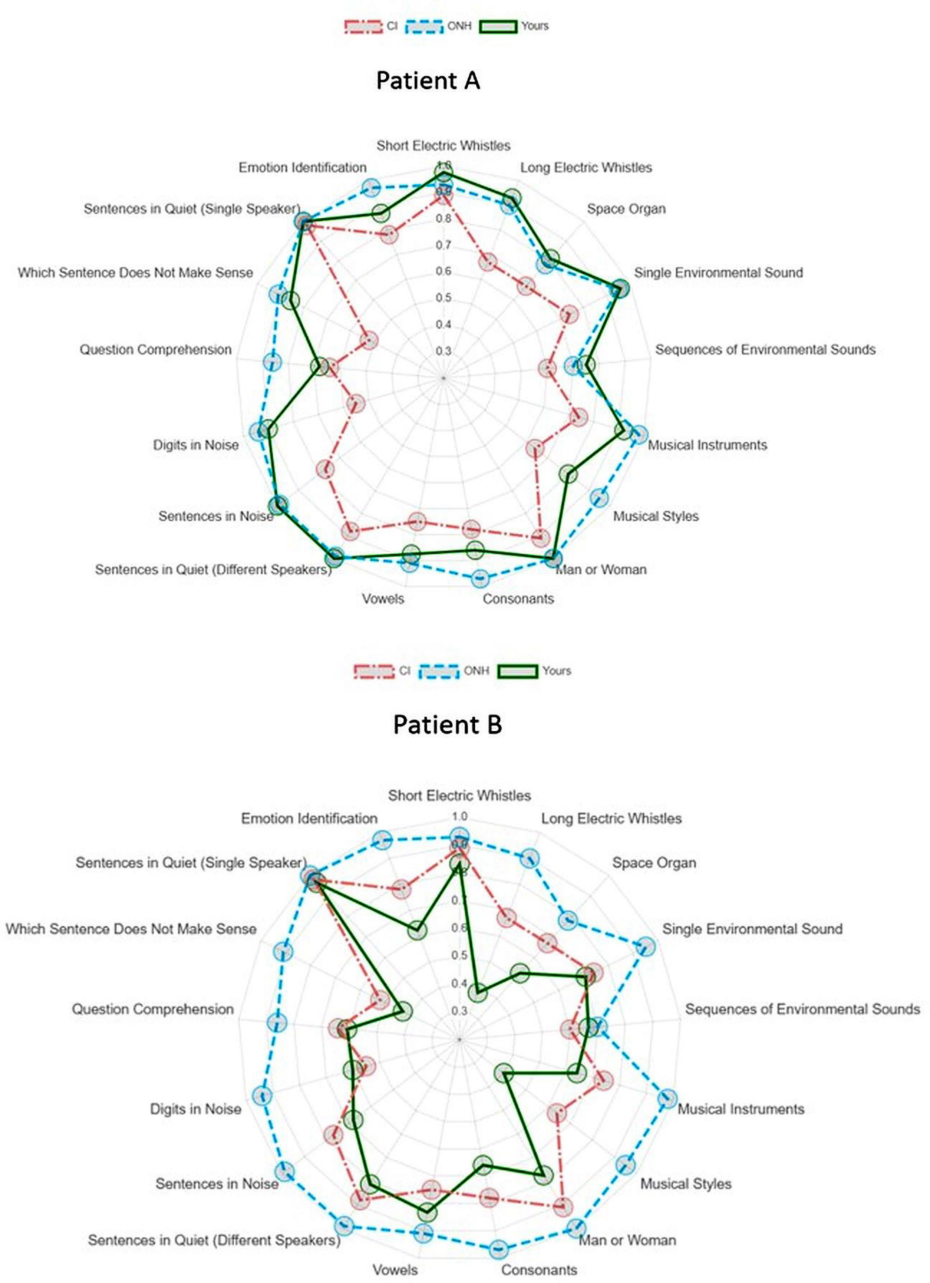
Performance of two CI users on the 17 tests of BASE battery shown as radar plots. Each patient’s performance on every test is contrasted with the average of CI users and older normal hearing (ONH) adults. The ONH and CI group performance and the tests of BASE battery are described in detail in Shafiro et al. (2020). BASE battery tests assess four broad categories of auditory abilities: (i) spectro-temporal processing (short and long “electric whistles,” i.e., stochastically modulated frequency patterns tracked on duration and SNR, respectively, and “space organ” i.e. spectral ripple discrimination), (ii) meaningful everyday sounds (identification of single environmental sounds and sound sequences, musical instruments and musical style identification), (iii) speech perception in quiet (male/female voice identification, vocal emotion identification, sentences in quiet [same and different speakers], sentences in noise [different speakers], identification of consonants and vowels in monosyllabic words), and (iv) speech perception in noise (sentences in noise, digits in noise, question comprehension, identification of semantically anomalous sentences).

Another benefit of remote testing is that it can expand the time available for testing beyond those of a clinical appointment. Patients can self-test in the comfort of their home and at the time of their choosing. With additional time, more tests can be performed to create a comprehensive profile of patient abilities and identify specific areas of strengths and weaknesses. Figure 2 illustrates an example of two comprehensive assessments for two patients on 17 tests of BASE battery. For each patient, A and B, a radar plot is generated that shows the profile of a patient’s performance on every test in relationship to the average CI group performance and that of older normal hearing peers based on results in Shafiro et al. (2020). Each radar plot provides a patient-specific profile of auditory abilities in different aspects of speech and auditory perception, which can provide a basis for patient tailored counseling and rehabilitation program. It can be seen that patient A’s performance generally approaches that of the average of older normal-hearing (ONH) peers and surpasses that of the CI group. In contrast, patient B’s performance does not exceed the CI group average on several tests and even falls below that on several tests. Based on these assessment results, different recommendations could be appropriate for patients A and B, after taking into account their individual needs and goals. Patient A may choose to focus on improving their ability to perceive vocal emotion and comprehension of the meaning of spoken utterances. In contrast, patient B may instead focus on improving aspects of sensory and basic linguistic encoding, training to discriminate voices, spectro-temporal auditory patterns, consonants, and practicing with digits in variable amounts of background noise. Depending on each patient’s preferences, specific rehabilitation recommendations can also extend beyond speech perception to include environmental sounds and music.

#### 4.2.2 Qualitative and ecological momentary assessment approaches

Qualitative and Ecological Momentary Assessment (EMA) approaches may also provide additional valuable insights into the real-world listening experiences of CI users. Qualitative interviews or focus groups can be designed to capture CI users’ communication needs, which may provide rich, contextual information for interpreting behavioral results. For example, qualitative studies on music perception in CI users have highlighted the challenges of processing pitch and timbre, as well as the strategies that CI users develop to improve musical enjoyment (Bartel et al., 2011; Gfeller et al., 2019). More broadly, qualitative approaches have shed light on CI users’ perceptions of the process of obtaining a CI, communication abilities post-CI, adaptation strategies, and the social–emotional impact of hearing loss in greater depth (e.g., Dillon and Pryce, 2020; Hornsby et al., 2021; Hughes et al., 2018; Hunniford et al., 2023; Khursheed et al., 2009; McRackan et al., 2019; Rapport et al., 2020; Rembar et al., 2009). Importantly, incorporating patients’ lived experiences into research can inform device programming and rehabilitative strategies for improved satisfaction and quality of life.

EMA is a tool that can be used to capture auditory experiences in real time. Using smartphone-based surveys or wearable devices, participants are prompted throughout the day to report on their device use, listening environments, and perceived communication success. By reducing reliance on retrospective recall, EMA provides an ecologically valid perspective on how CI users navigate the challenges of everyday communication. Further, EMA offers a way to connect CI users’ experiences to clinical speech perception scores. For example, O’Neill et al. (2021) used EMA to track everyday listening behaviors and social engagement in adult CI users and normal-hearing adults. They found that poorer performing CI users (on a sentence recognition test) spent more time at home and less time in conversation than higher performing CI users and NH adults. CI users also reported using compensatory strategies (e.g., visual cues), while NH adults did not. Similarly, research by Dunn et al. (2021) and Wu et al. (2022) used EMA to examine how COVID-19 pandemic measures impacted the listening environments of adult CI users, demonstrating that CI users experienced quieter listening environments and also reported better speech understanding, reduced listening effort, and less activity limitations. These findings suggest that CI users and NH adults may have qualitatively different communicative and social experiences, even when environmental access is similar. Such behavior patterns may help explain variability in long-term outcomes and offer new avenues for tailoring counseling and rehabilitation. As such, EMA addresses a major blind spot of conventional speech recognition tests: it captures what CI users actually do in the real world outside the clinic or research lab, not just what they can do in controlled conditions.

## 4 Discussion and conclusion

Speech perception testing has been instrumental in demonstrating the effectiveness of CIs and has been firmly embedded in clinical guidelines and candidacy criteria. As such, it has enabled hundreds of thousands of patients to regain functional hearing and improve aural communication. However, the vast new knowledge about speech perception mechanisms in normal, impaired and electric hearing accumulated since the early days of CIs has had limited penetration into clinical practice. Although potentially discouraging, this is hardly a new predicament. As early as 1984, when CIs were just beginning to be recognized and accepted as a viable treatment option for adults with SNHL, Walden (1984) in his introduction to an edited volume on speech recognition by the hearing impaired remarked on the slow adaptation of novel tests. Specifically, he wrote “In recent years, several new test materials, methodologies, and analyses have been suggested. Yet, due to a variety of limitations, none have received widespread acceptance. As a result, despite our substantially increased knowledge of speech recognition by the hearing impaired, old testing approaches largely persist. Although this may be disturbing, it is probably quite predictable. In the absence of a universally acceptable alternative, and faced with the necessity of doing something, clinicians and researchers are likely to resort to old, familiar approaches despite their obvious limitations.” More than 40 years later, Walden’s words continue to ring true, being as applicable to the present moment.

Our theoretical understanding of the intricacies of speech perception has considerably broadened over the last half century, resulting in new comprehensive information-processing models that address interactions of sensory bottom-up and cognitive top-down factors (Pichora-Fuller et al., 2016). In parallel to that, breakthroughs in electronic speech recognition and synthesis have made them commonplace technologies in everyday communications. These advancements have in turn been accompanied by the development of numerous novel tests and training materials that can be used to assess and modify more specific aspects of speech perception such as accents and dialectal variation, effort, context and listener experience. To date, few of these newly developed tests are routinely utilized in clinical settings, and as illustrated in Figure 1, the original monosyllabic word test, CNC, has remained most common in assessing performance of CI users. But there is also progress -- CNC has been closely followed by AzBio sentence test, which is the second most common, and has been developed in later years to include a variety of talkers and difficulty levels through the use of variable SNRs of multitalker babble.

Over the last decade, both tests, CNC and AzBio, have become the most widely used in clinical settings. Although many factors play a role in the choice of a clinical speech perception test, the successful adaptation of AzBio and the continuing reliance on CNC in CI clinics in North America, may reflect their complementarity and practical utility. Monosyllabic words in quiet with no additional context can indicate the integrity of bottom-up sensory integration and low-level phonemic-lexical encoding, while complex AzBio sentences, especially when delivered in noise, can provide an estimate of speech perception in more challenging real-world conditions. Both tests have a considerable range of difficulty levels that encompass the performance levels of most patients, and can thus be effectively used for establishing candidacy and outcome monitoring. On the other hand, the two tests are less useful in revealing specific challenges that individual CI users encounter in everyday communication environments and informing programming adjustments or rehabilitation targets.

As our understanding of speech perception mechanisms and CI outcomes evolves, we must keep asking: Do our speech perception tests capture the complexity of real-world speech communication, and meet the diverse priorities and needs of adult CI users? It is highly unlikely that any single test can satisfy all demands. However, some tests may better align with patient-specific goals. Currently, no common integrated theoretical framework exists that guides selection of specific tests and their interpretation (Pisoni et al., 2008). Being aware of the limitations of specific tests can ensure that optimal tests are chosen, while the choice of a speech perception test should be based on assessment goals and informed by patient-specific communication needs. For example, updates to MSTB battery can include brief tests that also tap into broader linguistic and cognitive aspects of speech perception, as well as additional PROMs that reflect self-reported benefits. In addition, comprehensive remote batteries, such as BASE battery or similar, could be used for augmenting MSTB protocols with self-administered assessments that profile multiple aspects of auditory and cognitive functioning that are not captured by MSTB. These additional tests could guide selection of individualized rehabilitation targets.

Future research should prioritize assessments that bridge the gap between clinical and laboratory-based measures and everyday communication experiences. Priority should be given to the development and validation of new measures that also satisfy the following characteristics: (1) assess patient-specific communication needs, (2) are quick and easy to administer with no or minimal assistance from a clinician, and (3) provide actionable recommendations for improving patient-specific outcomes. Although the demands of clinical settings constrain the number and length of testing that can be completed, new online technologies enable patient testing in their unique communication environments, potentially providing more accurate and individually-tailored results. However, to be widely adopted, new tools must undergo rigorous validation, including demonstration of reliability, sensitivity to change, and clinical utility. Future CI rehabilitation may also be further enhanced by speech perception training that can be tailored to specific patients’ communication needs. For instance, the ability to quickly synthesize speech materials based on a given speaker’s vocal transfer function can result in fast and inexpensive development of a patient-specific sets of training utterances targeting the voices of conversational partners the patient is most interested in communicating with and their specific dialectal preferences (Kadam et al., 2021; Azzuni and El Saddik, 2025). Indeed, past research indicates that speech perception training using the utterances produced by talkers with whom patients communicate most frequently leads to greater perceived benefit (Tye-Murray et al., 2016). Furthermore, interactive electronic conversational agents, already ubiquitous in daily life (e.g., smartphones, Alexa, Siri), may be leveraged as rehabilitation platforms for dynamic conversational activities, thus combining speech perception and production training customizable to patient-specific needs. By integrating behavioral speech perception tasks, cognitive-linguistic assessments, PROMs, and qualitative methods, research can gain a holistic view of CI users’ everyday communication experiences, abilities, and needs. These advancements will not only enhance the assessment of real-world CI outcomes but also inform individualized intervention strategies that optimize communication success and quality of life for adult CI users.

## Footnotes

^1^ www.basebattery.org
